# Development of an Electrochemical Biosensor to Detect miRNA Encapsulated in Lipid Nanoparticles

**DOI:** 10.1021/acs.analchem.5c00692

**Published:** 2025-05-24

**Authors:** Wanda Cimmino, Alessia Angelillo, Giuseppina Rea, Panagiota M. Kalligosfyri, Valeria Nele, Virginia Campani, Giuseppe De Rosa, Stefano Cinti

**Affiliations:** † Department of Pharmacy, 9307 University of Naples Federico II, 80131 Naples, Italy; ‡ Department of Life Science, Health and Health Professions, 207131 Link Campus University, Rome 00165, Italy; § Bioelectronics Task Force at University of Naples Federico II, Via Cinthia 21, Naples 80126, Italy; ∥ Sbarro Institute for Cancer Research and Molecular Medicine, Center for Biotechnology, College of Science and Technology, Temple University, Philadelphia, Pennsylvania 19122, United States

## Abstract

Lipid nanoparticles (LNPs) represent a versatile delivery platform proposed for a wide range of therapies based on nucleic acids, including microRNA (miRNAs). The ability of LNPs to encapsulate and protect RNA from degradation, as well as their ability to promote cellular uptake, has led to their clinical use with the approval of RNA-based medicinal products, i.e., COVID vaccines. In this context, a growing number of LNP formulations with improved transfection and biocompatibility are under development, requiring rapid, sensitive, and robust quality control tests, e.g., for the quantification of the encapsulated RNA. Nowadays, classical analytical approaches such as fluorescence, ultraviolet–visible (UV–vis) spectrophotometry, and chromatography are mainly used for the quantification of the encapsulated drug. However, the user-friendly and cost-effective quantification of the encapsulation efficacy within LNPs represents an important research focus, as it would allow monitoring of the amount of encapsulated RNA, thus providing immediate quality control. In this work, we present the adaptation of an electrochemical strip to quantify the encapsulation of a miRNA, i.e., miR-218, whose antitumor effect has been widely reported in the literature within LNPs. We provide a rapid and sensitive method to assess the concentrations of miRNA actually encapsulated, obtaining satisfactory agreement compared to the traditional fluorimetric approach. Specifically, the platform is based on a commercial gold-screen-printed electrode modified with a DNA probe designed to be fully complementary to the target miRNA-218. The electrochemical system was successfully combined with a 3D-printed chamber that allowed the use of multiple electrodes simultaneously and the use of Triton X-100 surfactant to disrupt the LNPs and release the encapsulated miRNA-218 achieving a detection limit as low as 1 nM.

## Graphical abstract

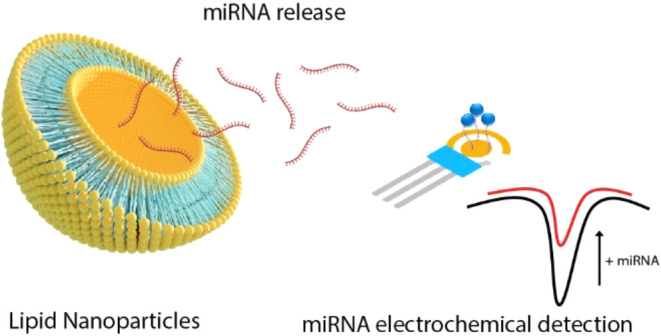

## Introduction

RNA-based therapies have emerged as a revolutionary approach in the field of biomedicine, capable of addressing many unmet medical needs by current treatments.^1^ These therapies provide the potential to manipulate gene expression or produce therapeutic proteins, making them particularly suitable for diseases with well-defined genetic targets, including infectious diseases, cancers, immune disorders, and Mendelian disorders such as neurological conditions.^2^ Despite being promising, the clinical use of RNA-based medicines is hampered by biopharmaceutical challenges. The relatively high molecular weight of therapeutic RNAs, their anionic charge, and their susceptibility to RNases in the bloodstream and tissues hinder their efficient cellular uptake and overall therapeutic efficacy.^3^ In this context, various delivery systems have been developed to protect RNA from degradation, maximize delivery to target cells, and minimize exposure to off-target cells.^2^

Among nonviral vectors, lipid nanoparticles (LNPs) are currently considered the gold standard platform for RNA delivery, with three distinct products approved for human use: mRNA-based vaccines, namely, Comirnaty and Spikevax, along with Onpattro (patisiran), the first RNA interference (RNAi) therapeutic approved.^1^ Given the ongoing focus on LNPs by research teams and pharmaceutical companies in the development of next-generation RNA-based therapeutics, the accurate detection and quantification of encapsulated RNA have emerged as critical aspects of this field. These processes are essential for assessing encapsulation efficiency, optimizing therapeutic dosing, and ultimately ensuring the efficacy and safety of RNA-based medicines.^4^

Nowadays, two commonly employed methods for RNA quantification are spectrophotometric analysis and the RiboGreen assay, which is a fluorescence-based method. However, these analytical methods often require multiple steps, mainly involving the use of solvents and centrifugation, and specialized equipment and personnel.^5^

Therefore, more streamlined and accessible approaches tailored to the specific requirements of RNA detection in therapeutic formulations for real-time monitoring are needed.

This is where the need for alternative detection tools like electrochemical biosensors arises due to the importance of their numerous benefits of the rapid, cost-effective, and highly sensitive approach^6^ to detect RNA encapsulation. Furthermore, electrochemical biosensors provide versatility, high specificity, and sensitivity for RNA detection, as reported by several research groups.^7–10^

In a recent work of our group, we demonstrate the applicability of the electrochemical sensors to detect the cargo of the lipid nanovectors utilizing a redox mediator, namely, methylene blue (MB), as cargo.^11^ Starting from this point, we propose, for the first time, an electrochemical biosensor to quantify miR-218 in LNPs, whose antitumor effect has been widely reported in the literature^12,13^ and here used as a model RNA sequence. The biosensor is based on a screen-printed gold electrode functionalized with a specific single-stranded (ss)-DNA probe, enabling the selective detection of miR-218 in LNP-based matrices. Triton X-100 surfactant was used to disrupt the LNPs^14^ and release the encapsulated miRNAs, following their accurate quantification with a detection limit down to 1 nM. In addition, 3D-printed cells were designed to allow simultaneous measurements of multiple samples in the presence of Triton X-100. Notably, this electrochemical biosensor outperforms other spectrophotometric techniques in the assessment of miRNA levels and therefore can be considered a promising tool for therapeutic applications. Due to its relatively low cost (<3€ per electrode), quick assay performance, and absence of bulky equipment, it is highly useful in the field of therapeutics and quality assurance. Furthermore, compared to fluorimetric methods such as the Quant-iT RiboGreen RNA assay, which requires expensive kits and specialized fluorescence readers, this biosensor offers a more cost-effective and portable solution. Its ability to achieve detection limits at nanomolar concentrations makes it particularly advantageous for real-time monitoring of miRNA-containing LNPs, enabling precise quantification of the dosage administered to patients. This capability could pave the way for more effective and personalized therapies while advancing miRNA quantification techniques overall.

## Experimental Section

### Chemicals and Materials

All of the chemicals, PBS tablets (140 mM NaCl, 10 mM phosphate buffer, 3 mM KCl), 6-mercapto 1-hexanol (MCH, C6H14OS), tris­(2-carboxyethyl) phosphine (TCEP; C9H15O6P), sulfuric acid (H_2_SO_4_), Triton X-100, cholesterol (CHOL), sodium chloride, sodium citrate, citric acid, and ammonium ferrothiocyanate, were purchased from Sigma-Aldrich (St. Louis, MO). The ssDNA probe anti miR-218 (5′-thiol-C6-ACA TGG TTA GAT CAA GCA CAA-Atto MB2–3′) (MB-DNA probe), the miR-218 target (5′-UUG UGC UUG AUC UAA CCA UGU-3′), and the miRNA sequences utilized for the selectivity study miR −101–5p (5′-CAG UUA UCA CAG UGC UGA UGC U-3′), miR-125B-5p (5′-UCC CUG AGA CCC UAA CUU GUG A-3′), and miR-200A-5p (5′-CAU CUU ACC GGA CAG UGC UGG A-3′) were provided by Metabion GmbH (Steinkirchen, Germany). The miR-218 (5′-rUrUrGrUrGrCrUrUrGrArUrCrUrArArCrCrArUrGrU-3′) was synthesized by Tema Ricerca s.r.l. (Bologna, Italy). SM-102 (8-[(2-hydroxyethyl)­[6-oxo-6-(undecyloxy)­hexyl]­amino]-octanoic acid, 1-octylnonyl ester) was purchased by Cayman Chemical (Ann Arbor). DMG-PEG 2000 (1,2-dimyristoyl-rac-glycero-3-methoxypolyethylene glycol-2000) was provided by Avanti Polar Lipids (Alabaster). Disteroylphosphatidylcholine (DSPC) was kindly offered from Lipoid GmbH (Ludwigshafen, Germany, Switzerland). The Quant-iT RiboGreen RNA Assay was supplied from Thermo Fisher Scientific (Milan, Italy), while ethanol and other solvents were obtained by Exacta Optech (Italy).

The gold-screen-printed electrodes 220AT (WE and AUX/Au; REF/Ag) were obtained by a Metrohm DropSens. The electrochemical measurements were performed with a Palmsens multiemstat 4, 8 channels potentiostat (PalmSens, The Netherlands), and data were recorded using the software PSTrace 5.10 interfaced with a laptop. The 3D-printed cells were created by a Creality Ender-3 V2 Neo 3D printer (Shenzhen Creality 3D Technology, Shenzhen, China).

### Biosensor Fabrication on Screen-Printed Gold Electrodes

The first step of biosensor production was the pretreatment of the gold working electrode surface. Electrochemical activation of the working electrode surface was performed by applying multiple cyclic voltammetry cycles in 0.5 M of H_2_SO_4_ scanning from 0 to +1.5 V with a scan rate of 1 V/s and an E step of 1 mV. This step was critical for cleaning and H^+^ treating of the working area.^15,16^ The electrodes were then rinsed with deionized (DI) water and dried at room temperature. Once the gold surface was activated, the modification of the biosensor working electrode was performed according to a protocol optimized in our previous studies.^7,16–18^ In the first step of modification included the reduction of the S–S bond of the MB-DNA probe in the presence of 0.01 M TCEP for 1 h in the dark. After the reduction step, the probe was diluted in PBS to 100 nM and a 20-μL drop was applied to the working area, following an incubation step for 1 h in a humidity chamber. After this incubation time, the working area was gently rinsed with DI water, the working electrode was incubated in the humidity chamber in the presence of 2 mM of MCH for 1.5 h, and then the working electrode was gently washed with DI water. The miRNA target detection is based on its hybridization with the capture MB-DNA probe. The proposed biosensor is characterized as a signal-off platform because in the presence of the miRNA target in the solution its hybridization with the MB-DNA probe occurs, leading to a decrease of the MB reduction on the working electrode surface. This means that a lower current response will be recorded with respect to the blank measurement, i.e., the absence of a target. All of the results of this study are interpreted in terms of signal change % between the current recorded in the absence of the target solution and the current recorded in the presence of different concentrations of miR-218. Figure 1 shows the whole workflow, which includes miRNA encapsulation, miRNA release, thanks to the use of Triton X-100, and the electrochemical detection of the released miRNA.

**Figure 1:**
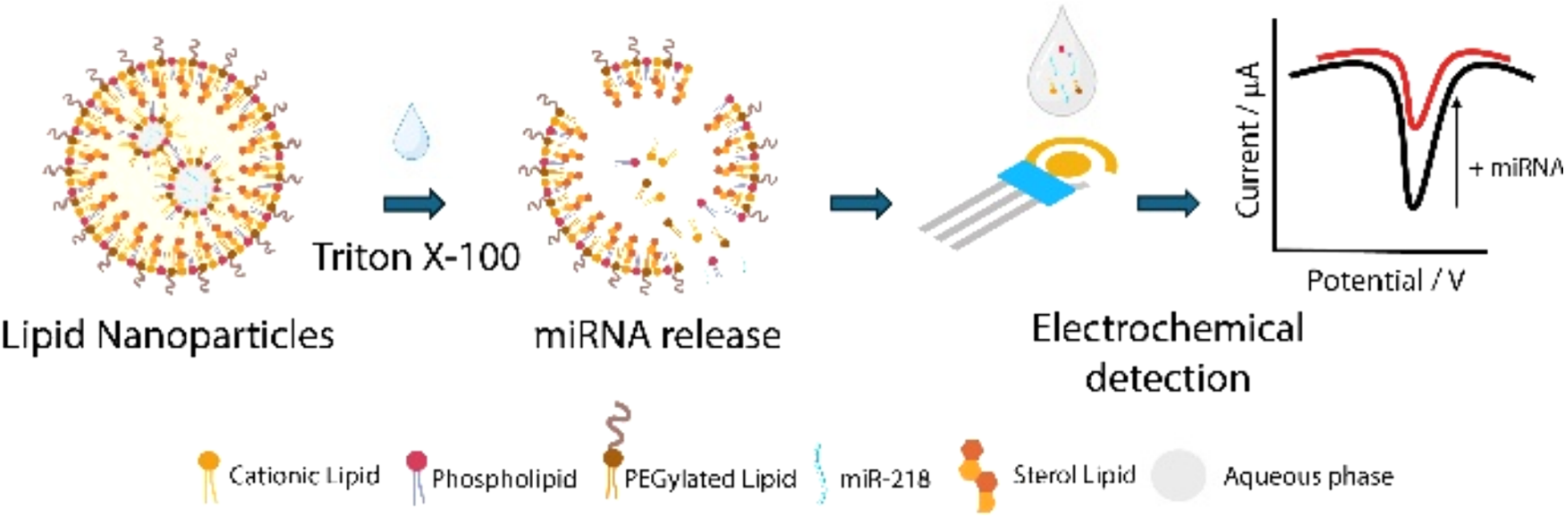
Workflow of the whole experimental setup.

### Electrochemical Measurements

Electrochemical measurements were performed at room temperature with up to eight electrodes inserted in a multichannel potentiostat and their immersion in the 3D-printed chambers, and the electrochemical measurements set up is reported in Figure S2, Supporting Information. All of the measurements were performed in the presence of Triton X-100, which was used to disrupt the LNPs and allow the release of miR-218. The measurements were performed using square wave voltammetry (SWV) with the following experimental parameters: T equilibration = 5s E begins = +0.1 V, E ends = −0.6 V, E step = 0.001 V, Amplitude = 0.01 V, and frequency = 50 Hz.

### Preparation of LNPs Encapsulating miRNA-218

LNPs encapsulating miRNA (LNP miRNA-218) were prepared using the ethanol injection method.^19^ Briefly, an ethanol stock solution composed of SM-102/CHOL/DSPC/DMG-PEG 2000 (50:38,5:10:1,5 mol %) and a miRNA-218 citric acid solution (10 mM, pH 4.0) were prepared. The lipid ethanol solution was then added dropwise to the buffer solution under stirring in a 1:1.5 v/v ratio (*N*/*P* = 6). The preparation was dialyzed (20 kDa cutoff) against 10 mM citrate buffer (pH 4.0 for 1 h) to remove the excess ethanol and then against PBS 1x pH 7.4 overnight to remove the citrate buffer and neutralize the LNP surface charge. LNPs were concentrated using Amicon filters with a molecular weight cutoff of 3.5 kDa. Empty LNPs were also prepared and used as control samples.

### LNPs Physicochemical Characterization

The formulations were characterized in terms of colloidal dimensions, polydispersity index (PDI), and surface charge using dynamic light scattering (DLS) (Zetasizer Nano Z, Malvern, U.K.). For each formulation, the z-average diameter, PDI, and zeta potential were calculated as the mean value ± standard deviation of the measurements from *N* = 3 independent batches. All of these results are reported in Figure S1, Supporting Information.

### Lipid Dosage in LNPs

The amount of phospholipids in the LNP miRNA-218 was determined by the Stewart assay.^20^ Briefly, an aliquot of the LNPs was added to a two-phase system consisting of an aqueous ammonium ferrothiocyanate solution (0.1 N) and chloroform. Each tube was mixed on a vortex and then centrifuged (Hettich UNIVERSAL 320 R, Andreas Hettich GmbH) at 1000 rpm for 10 min. The chloroform phase was collected, and the concentration of DSPC was obtained by measurement of the absorbance at 485 nm with an ultraviolet–visible spectrophotometer (UV VIS 1204; Shimadzu Corporation, Kyoto, Japan). The concentration of the total lipid content was calculated considering a constant ratio between the lipids.

### Assessment of Encapsulation Efficiency of the miR-218 in LNPs

The miRNA encapsulation efficiency (EE) was quantified by the fluorimetric assay using the RiboGreen RNA Quant-iT Assay kit (Thermo Fisher); for this purpose, the formulations are diluted in Tris-EDTA (TE) 1x buffer containing 1% (w/v) Triton X-100 (permeabilized LNPs) or TE 1x buffer (intact LNPs). A miRNA standard curve (R^2^ = 0.99) was used. The miRNA encapsulation efficiency was determined as follows

I $$\mathrm{EE}\left(\%\right)=\frac{{\left[\mathrm{miRNA}\right]}_{\mathrm{total}}-{\left[\mathrm{miRNA}\right]}_{\mathrm{unencapsulated}}}{{\left[\mathrm{miRNA}\right]}_{\mathrm{total}}}\times 100$$

where [miRNA]_total_ is the total miRNA concentration in the formulation and [miRNA]_unencapsulated_ is the concentration of unencapsulated miRNA.

## Results and Discussion

### Triton X-100 Optimization

Triton X-100 is a nonionic surfactant used to open LPNs to facilitate the release of encapsulated nucleic acids.^14^ In this study, it was used for the degradation of lipid structures to allow the release of the encapsulated miRNAs thus enabling the measurements. Of paramount importance is the optimization of the concentrations of Triton X-100 to be used for opening the LNPs; in fact, if the concentration of this detergent is too low, there is a risk of not achieving complete lipid degradation, resulting in the release of lower concentrations of nucleic acids than are actually encapsulated, if the concentration is too high, there is a risk of degrading the structure of the released nucleic acids.^21,22^ For these reasons, we decided to optimize the concentrations of Triton X-100 to be used in this study by testing the opening of the LNPs in the presence of different concentrations of Triton X-100. As shown in Figure 2, the optimal concentration to be used for LNP opening is 0.5% (w/v). In fact, employing this concentration, the highest signal change % was obtained with respect to the 0.1%, which is not sufficient for the complete opening of the LNPs, and with respect to the concentrations of 1 and 2%, which probably interact with the nucleic acids of the system degrading the released miRNAs and the DNA capture probe, leading to a lower signal change% in the presence of the miRNA target.

**Figure 2:**
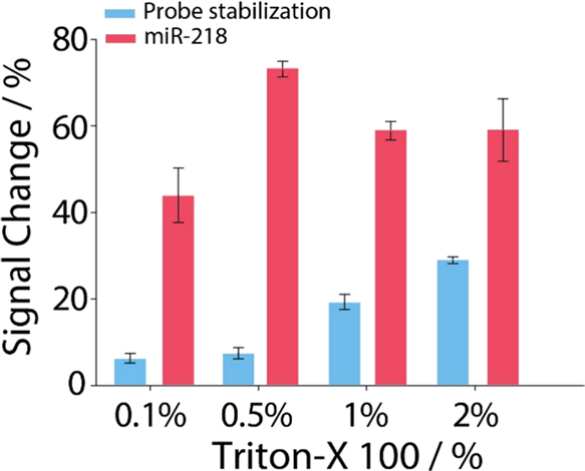
Optimization of Triton X-100 concentrations ranging from 0.1 to 2% w/v, used for the opening of miRNA-loaded LNPs. All of the measurements were performed in triplicate.

Indeed, at a Triton X-100 concentration of 0.5%, we observed the highest signal change%, compared to both the lower concentration (0.1%), which appears insufficient for complete LNP disruption, and the higher concentrations (1 and 2%), which likely interact with nucleic acids in the system. These higher concentrations may contribute to the degradation of both the released miRNAs and the DNA capture probe, ultimately resulting in a reduced signal change% in the presence of the miRNA target.

Our hypothesis regarding the interaction of Triton X-100 with the immobilized DNA probe is further supported by the probe stabilization data obtained using empty LNPs. As shown in Figure 2 (blue histograms), which represent signal change% in the absence of the target miRNA, the highest concentrations of Triton X-100 resulted in an increased signal change after the stabilization period. This suggests that Triton X-100 compromises the probe stability, leading to ongoing signal loss.

Conversely, at lower Triton X-100 concentrations (0.1 and 0.5%), the signal change% remains around 5%, indicating that the probe remains stable under these conditions. The improved signal change observed in the presence of the target miRNA (Figure 2, red histograms) under these conditions is thus likely due to a combination of the reduced background signal from the blank sample and greater stability of the immobilized probe at lower Triton X-100 concentrations.

### Analytical Performances, Selectivity Study, and miRNA Quantification

To evaluate the analytical performance of the electrochemical platform, the binding curves were evaluated in buffer solution (140 mM NaCl, 10 mM phosphate buffer, 3 mM KCl pH 7.4) and in spiked empty LNPs. The working solutions were spiked with different concentrations of miR-218 ranging from 1 pM to 800 nM. As shown in Figure 3, a characteristic semilogarithmic sigmoidal correlation was observed between the signal change % and the logarithmic scale of the target concentration expressed in nanomolar. Importantly, this sigmoidal trend was evident in both buffer solution and LNP measurements, confirming the robustness and versatility of the method in different matrices. In addition, a high correlation of 0.98 was observed for miR-218 in both solutions. The LOD was determined by identifying the miR-218 concentration at which the signal reached 10% of the maximum response recorded in the saturation region.^23^ Based on this method, the LOD was found to be approximately 0.45 nM in the buffer solution and 1 nM in the real matrix. The repeatability of the platform was evaluated by calculating the relative standard deviation (RSD%) and was found to be less than 5% (*n* = 6) in both cases, confirming the robustness of the platform toward real matrices testing.

**Figure 3:**
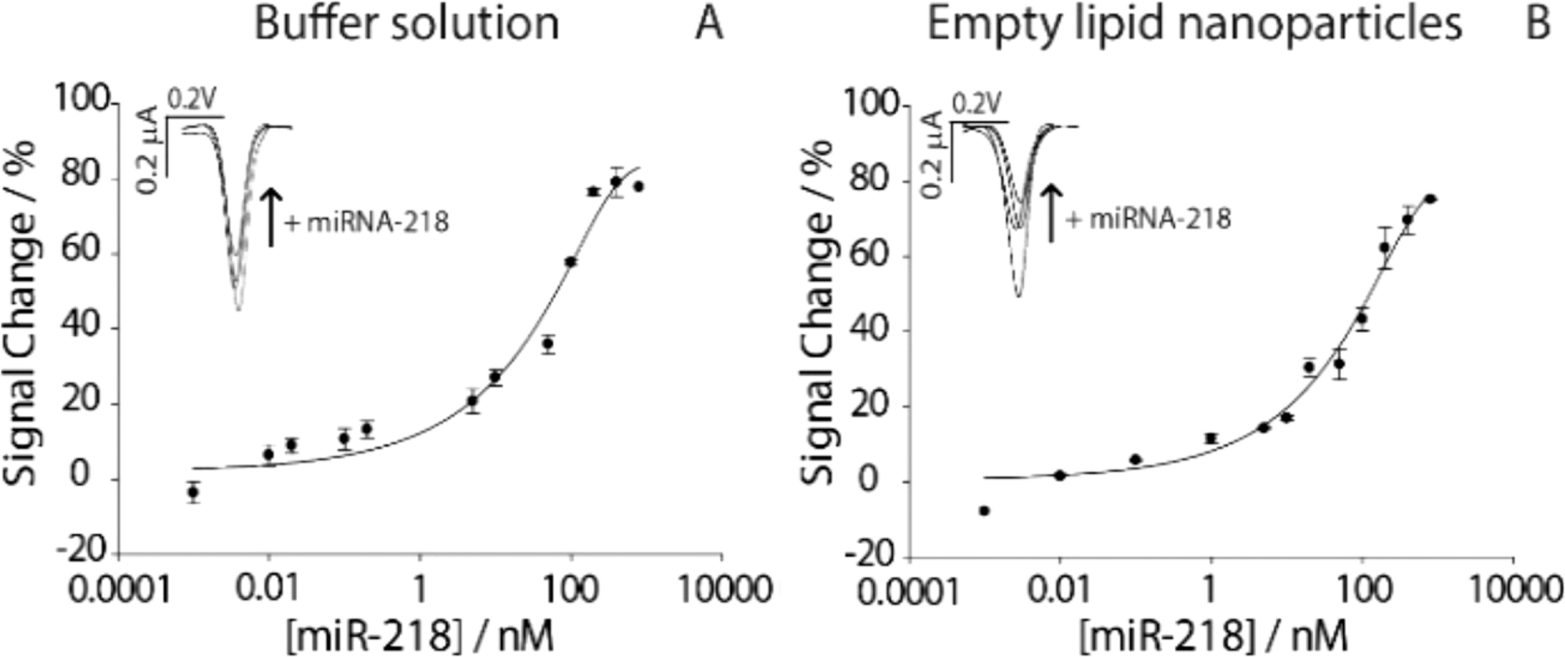
Binding curves obtained by testing different concentrations of miR-218 ranging from 1 pM to 800 nM. (A) Binding curve obtained in buffer solution. (B) Binding curve obtained in LNPs. The insets of each figure show the signal off voltammetric curves corresponding to the increase of miR-218. All of the measurements were performed in triplicate.

To test the selectivity of the biosensor, the platform’s performance was evaluated in buffer solution in the presence of 20 nM of different miRNA sequences related to triple-negative breast cancer. Specifically for this study, the device was tested in the presence of three random miR sequences: miR-101–5p, miR-125B-5p, and miR-200A-5p, and the results were compared with the detection of 20 nM of the miR-218. As can be seen in Figure 4, the presence of interfering species was only responsible for negligible signal changes, lower than 15% against the signal change obtained by the miRNA target higher than 25%. To further validate these findings, an ANOVA followed by Tukey’s HSD test (FWER = 0.05) was performed on the interference study results. The statistical analysis demonstrated that the signal variation observed for miR-218 was significantly different from that obtained from the interferents (*p*-values <0.001), while no statistically significant differences were found among the interferent groups. These results further support the selectivity of this platform, confirming that the observed signal variation is specifically attributed to miR-218 rather than nonspecific interactions. Moreover, the absence of significant differences among the interferents reinforces that the sensor does not respond indiscriminately to other sequences, highlighting its ability to reliably distinguish miR-218 from similar miRNAs.

**Figure 4:**
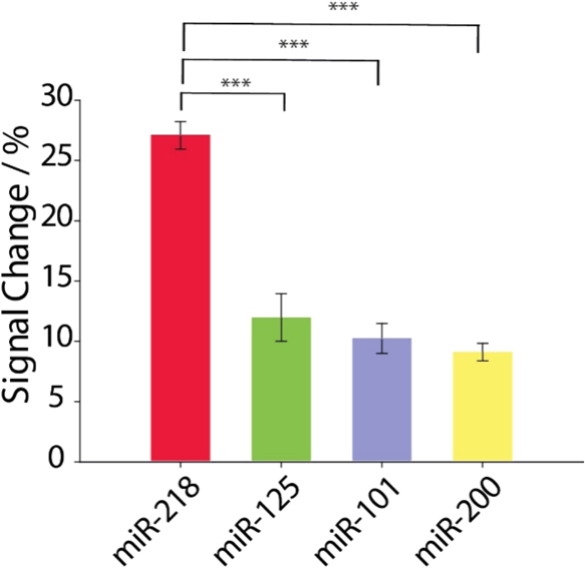
Selectivity study performed in buffer solution, comparing the signal intensities obtained in the presence of 20 nM of different miRNA sequences: red, miR-218; green, miR-125B-5p; violet, miR-101–5p; and yellow, miR-200A-5p. All of the measurements were carried out in the presence of 0.5% (w/v) of Triton X-100 and in triplicate. Statistical analysis was conducted using ANOVA followed by Tukey’s HSD test (FWER = 0.05). Horizontal lines indicate significant differences between groups: ***p < 0.001, **p < 0.01, and *p < 0.05.

Once the analytical performances of the platform were evaluated, the miRNA cargo in the LNPs was quantified. In particular, the electrochemical quantification was compared with the fluorimetric one. For the quantification of miRNA encapsulated in LNPs, different concentrations of loaded miRNA in LNPs were tested. The tested concentrations were 10, 50, 100, and 500 nM. Figure 5 shows a comparison between spiked empty LNPs and the loaded LNPs. The concentrations detected by the two methods are 17.9 ± 0.9 and 19.7 ± 3.0 μM for the electrochemical and the fluorimetric detection, respectively. Thus, it can be said that with the method proposed in this work, we were able to achieve an accuracy of 94% in the detection of encapsulated miRNAs.

**Figure 5:**
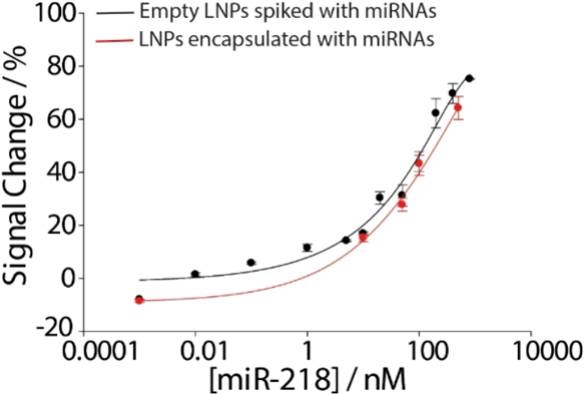
Comparison between the calibration curves obtained for empty spiked LNPs (black) and for miRNA-loaded LNPs (red). All of the measurements were performed in the presence of 0.5% w/v Triton X-100 and in triplicate.

## Conclusions

In this study, we successfully developed an electrochemical biosensor for the quantification of miR-218 encapsulated in LNPs. The platform is based on a screen-printed gold electrode modified with a specific ssDNA probe, enabling selective detection of the miR-218.

The electrochemical platform demonstrated fast and sensitive detection capability, with a detection limit of 1 nM, in the LNPs matrix. The detection limit appears to be well below the miRNA concentrations normally found in pharmaceutical formulations,^24^ confirming the platform’s applicability to real samples. Given its low cost, speed, and simplicity of design, this electrochemical biosensor represents a promising alternative tool for real-time dosage monitoring and quality control of RNA-based therapeutic drugs.

Future work will focus on further validating the biosensor in a clinical setting and exploring its application for other miRNAs and therapeutic formulations. This approach could significantly improve the efficiency of RNA delivery systems and contribute to the advancement of RNA-based therapies in clinical practice.

## Supplementary Material



## Notes

Characterization of LNPs and the setup of electrochemical measurements in 3D-printed cells (PDF)
