# Supplementary material for: Development of an Electrochemical Biosensor to Detect miRNA Encapsulated in Lipid Nanoparticles
Source: Anal Chem. 2025 May 24;97(23):11968–73. doi: 10.1021/acs.analchem.5c00692 (PMC12177871; doi:10.1021/acs.analchem.5c00692)
Supplement: Supplementary file 1 [file ac5c00692_si_001.pdf]

Development of an electrochemical biosensor for miRNA detection encapsulated in lipid nanoparticles

Wanda Cimmino[a‡], Alessia Angelillo[a‡], Giuseppina Rea[a], Panagiota M. Kalligosfyri[a], Valeria Nele[a], Virginia Campani[b], Giuseppe De Rosa[a], and Stefano Cinti[a,c,d*]

a Department of Pharmacy, University of Naples “Federico II”, 80131 Naples, Italy.
b Department of Life Science, Health and Health Professions, Link Campus University, Rome 00165, Italy
c Bioelectronics Task Force at University of Naples Federico II, Via Cinthia 21, Naples 80126, Italy.
d Sbarro Institute for Cancer Research and Molecular Medicine, Center for Biotechnology, College of Science and Technology, Temple University, Philadelphia, PA 19122, USA
*** Corresponding author: Stefano Cinti, stefano.cinti@unina.it**

Contents

Abstract	S1
Characterization of lipid nanoparticles	S1
Set-up of electrochemical measurments in 3D printed cells	S1
Comparison between the costs of the two quantification methods	S2

Abstract

The information regarding the lipid nanoparticles’ characterization, the set-up of the electrochemical measurements in the 3D-printed cells and the comparison between the costs of the two methods are reported here.

Characterization of lipid nanoparticles

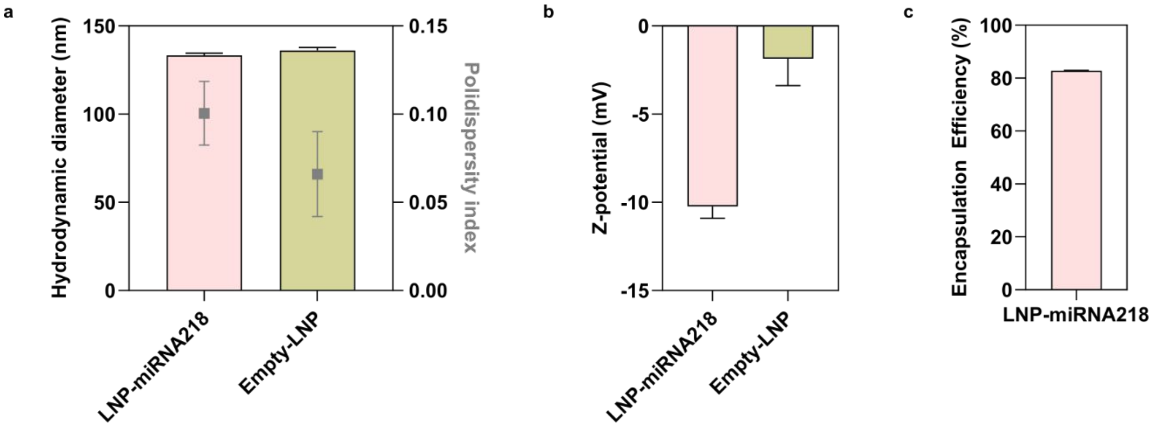

Figure S1: Average diameter and PDI (a), zeta potential (b), and mRNA encapsulation efficiency (c) of LNP encapsulating miRNA 218 and empty LNP.

Set-up of electrochemical measurements in 3D printed cells

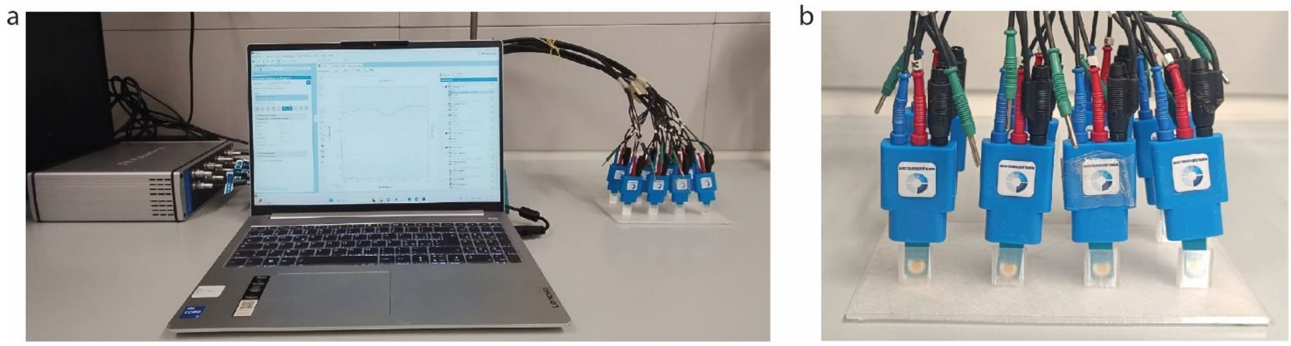

Figure S2: Electrochemical measurements set-up (a), 3D printed cells using transparent PLA filament (1.75 mm, R3D company) enabling up to 100 µL volume (b).

Comparison between the costs of the two quantification methods

Table S1: Cost comparison between the two methods used for the quantification of miRNA encapsulated in LNPs.

Parameter	Electrochemical Biosensor	Quant-iT RiboGreen
**Unit Cost**	0.20 - 3 € per electrode[1]	5-10€ per test[2]
**Required Equipment**	Potentiostat (<1k €)[3]	Fluorimeter (>10k€)[4]

References

(1) Kalligosfyri, P. M.; Miglione, A.; Cinti, S. Screen-Printing and 3D-Printing Technologies in Electrochemical (Bio)Sensors: Opportunities, Advantages and Limitations. *ECS Sens. Plus* **2025**, *4* (1), 010601. https://doi.org/10.1149/2754-2726/ada395.
(2) *Quant-it™ RiboGreen Reagent and RNA Assay Kit*. https://www.thermofisher.com/order/catalog/product/R11490 (accessed 2025-04-22).
(3) https://www.palmsens.com/product/sensit-smart/
(4) *Qubit™ 4 Fluorometer, with WiFi*. https://www.thermofisher.com/order/catalog/product/Q33238 (accessed 2025-04-22).
